# Deciphering GB1’s Single Mutational Landscape: Insights from MuMi Analysis

**DOI:** 10.1021/acs.jpcb.4c04916

**Published:** 2024-08-08

**Authors:** Tandac F. Guclu, Ali Rana Atilgan, Canan Atilgan

**Affiliations:** Faculty of Natural Sciences and Engineering, Sabanci University, Tuzla, Istanbul 34956, Turkey

## Abstract

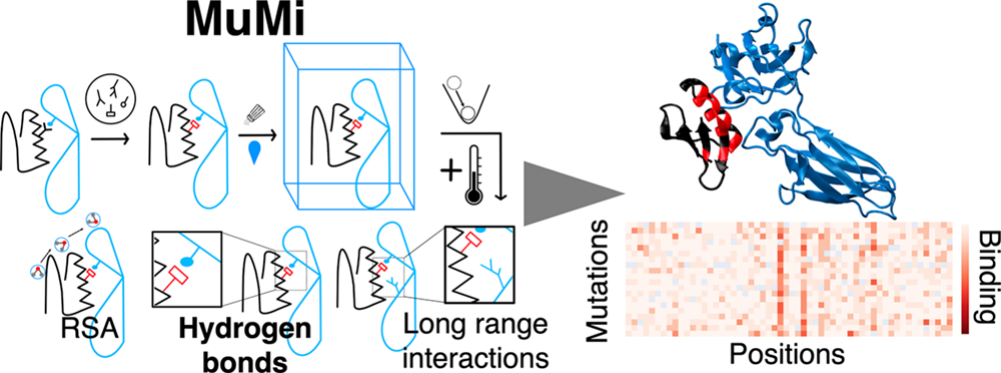

Mutational changes that affect the binding of the C2 fragment of Streptococcal protein G (GB1) to the Fc domain of human IgG (IgG-Fc) have been extensively studied using deep mutational scanning (DMS), and the binding affinity of all single mutations has been measured experimentally in the literature. To investigate the underlying molecular basis, we perform in silico mutational scanning for all possible single mutations, along with 2 μs-long molecular dynamics (WT-MD) of the wild-type (WT) GB1 in both unbound and IgG-Fc bound forms. We compute the hydrogen bonds between GB1 and IgG-Fc in WT-MD to identify the dominant hydrogen bonds for binding, which we then assess in conformations produced by Mutation and Minimization (MuMi) to explain the fitness landscape of GB1 and IgG-Fc binding. Furthermore, we analyze MuMi and WT-MD to investigate the dynamics of binding, focusing on the relative solvent accessibility of residues and the probability of residues being located at the binding interface. With these analyses, we explain the interactions between GB1 and IgG-Fc and display the structural features of binding. In sum, our findings highlight the potential of MuMi as a reliable and computationally efficient tool for predicting protein fitness landscapes, offering significant advantages over traditional methods. The methodologies and results presented in this study pave the way for improved predictive accuracy in protein stability and interaction studies, which are crucial for advancements in drug design and synthetic biology.

## Introduction

Deep mutation scanning (DMS) is a powerful technique used in the comprehensive analysis of the functional consequences of amino acid changes.^1^ With DMS, mutations are systematically introduced at every position in the protein and the effects of these changes are measured. However, DMS can be resource intensive and costly, especially when performed on a large scale or with multiple iterations. The limitations in DMS and the potential impact of single mutations on protein function to trigger many discoveries in the biochemical sciences has driven researchers to develop the relatively inexpensive computational methods^2^ whereby algorithms to analyze and interpret large-scale data generated by DMS experiments are developed.^3^ These approaches play a critical role in extracting meaningful insights from the vast amounts of sequencing data generated by DMS. Computational tools are used to predict the functional effects of amino acid substitutions based on features such as protein sequence, structure and evolutionary conservation.^4−7^ These tools aim to identify possible mutations that could alter protein function, stability or interactions.^8−10^ This approach includes identifying mutations that significantly alter protein function by comparisons with the wild-type (WT) sequence, mapping functionally important spots, and regions of structural importance. Computational methods integrate protein sequences as well as structural biology data such as protein structures and molecular dynamics (MD) simulations.^11^ Web servers and databases created as a result of these efforts provide tools and resources for analyzing DMS data; see e.g.^12^

Pathogenic missense variants disrupt protein function and reduce the fitness of the organism, while benign missense variants have limited impact. The AlphaMissense platform is an adaptation of AlphaFold (AF), fine-tuned on human and primate variant population frequency databases, to predict missense variant pathogenicity^2^ and discriminate pathogenic variants. In a similar missense prediction study, the RaSP method was developed to examine variants observed in the human population by calculating approximately 230 million stability changes for almost all single amino acid changes in the human proteome.^12^ For example, in AlphaFold single variant predictions, no significant correlation was found between output metrics and fitness values such as protein stability or fluorescence change.^13^ While missense variants are related to mutations observed in the clinic, DMS reveals the fitness of all single mutants, whether they exist in a natural environment or not. For this more comprehensive problem, applying artificial neural networks to DMS data has enabled protein sequence-function association.^14^ In one comprehensive study, hierarchical clustering was performed using the fitness values of 6291 positions in 30 proteins with DMS findings clustering the functions of amino acids in the three-dimensional structures of proteins.^15^ Models predicting allosteric regions using machine learning have also been proposed.^16^

While sufficient data is provided by DMS experiments, information on structural details lags far behind.^3^ Methods that account for external effects such as water and salt are required to track the small but significant changes in protein structure caused by point mutations in sufficient detail. Foremost among these are MD simulations and related advanced simulation techniques, which require gross computational time.^17^ The use of data from MD simulations in combination with machine learning techniques to overcome the lack of structural information has only recently been used.^14^

The processes contributing to the cumulative effects observed in DMS experiments can be calculated in a way that decouples folding and binding processes.^18,19^ On the down side, although mutation effects have been quantified and calculated as energy differences compared to the WT protein, the underlying molecular mechanisms leading to these values have not been identified. Previous efforts to find an atomic-scale link in the mechanisms leading to changes in conformational values in DMS have shown that the solvent accessible surface is informative about mutational effects on the binding affinity of hydrogen bonds and salt bridges.^19−22^ In particular, the classification of residues based on solvent accessibility^19,20,22^ without any measurement of binding energies shows promise. However, methods to predict the stability of GB1 using these factors as attributes in machine learning algorithms have fallen short of predicting the experimental findings to the desired degree.^19,20,22^

**Figure 1:**
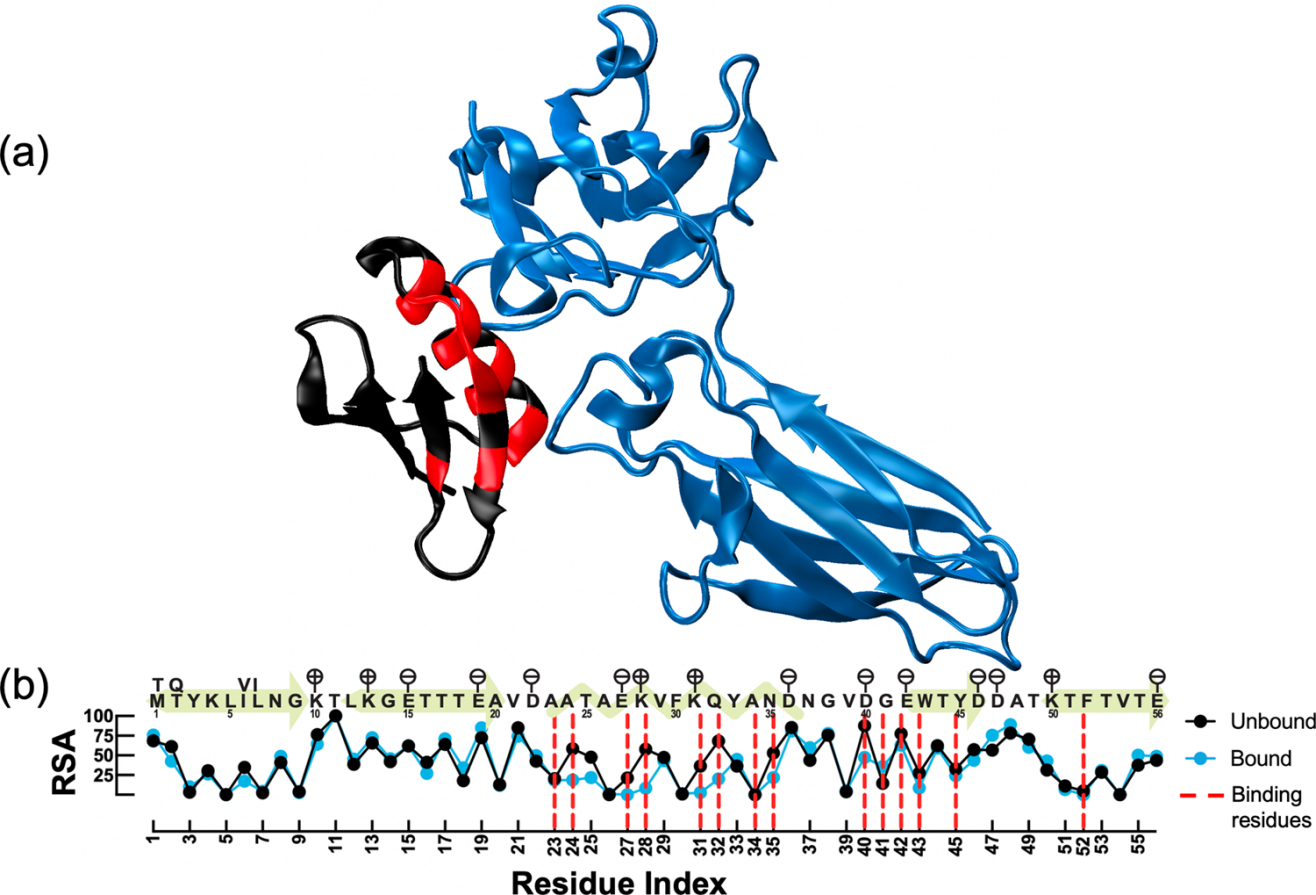
C2 fragment of Streptococcal protein G (GB1, in black/red) partnered with the FC domain of human IgG (IgG-Fc, in blue). (a) WT form of the complex is employed for visualization and analysis by using minimized 1FCC PDB-coded structure. GB1 and IgG-Fc have 56 and 206 residues, respectively. Binding region of GB1 (residues that have at least one heavy atom within 5 Å of IgG-Fc) is shown in red. (b) Sequence of GB1 is illustrated by signifying secondary structure (yellow patterns) and charged residues (flags above positions). The amino acid differences between the sequences of 1PGA (unbound GB1) and 1FCC (bound form) are noted for residue positions 1, 2, 6, and 7. Accompanying RSA is calculated for unbound/bound forms of GB1 after minimization in water. Residues at the binding region are indicated by red dashed lines. The solvent accessibility of residues 24, 25, 27, 28, 31, 32, 35, 40, and 43 decreases after the binding, while a slight increase occurs for residues 19, 41, 47, and 48. There are 9 hydrogen bonds between GB1 and its partner in minimized WT. Residues 27 and 28 have two hydrogen bonds per position, and residues 35, 39, 40, 42, and 43 have one each.

As a model system, the binding profile of all possible single and double mutations belonging to C2 fragment of Streptococcal protein G (GB1) and Fc domain of human IgG (IgG-Fc) have been measured experimentally (see Figure 1 for structures).^23^ Furthermore, several publications discussed the effects of these mutations by changing up to four positions in the sequence^24^ and by stability experiments.^25,26^ GB1 was also used as a case study to predict protein structures by only using epistatic relations between residue pairs.^27,28^ The processes contributing to the observed cumulative effects in the DMS experiments were separated into those due to binding fitness and folding fitness.^18,19^ Although the effects of mutations are measured with respect to the WT and calculated as energy differences, the underlying molecular mechanisms leading to these values are not well-established.^23−25,29,30^

Aiming to fill the knowledge gap, we have scrutinized the conformations and the dynamics of GB1 for unbound and bound forms by using computational methods. In particular, we seek to understand the degree to which changing the side chain followed by minimization in the correct solvent environment describes the shifts in the average positions of all atoms of the protein. This approach finds root in previous work where we have shown that in proteins, local displacements in the three-dimensional structure that disturb hard degrees of freedom, such as bond lengths, are not restored to their initial coordinates upon minimization but are instantaneously perpetuated to the whole structure through the rearrangements of soft degrees of freedom, such as torsions and nonbonded contacts.^31^ In fact, this idea leads to an efficient conformational search method for small molecules.^32,33^ The so-called mutation-minimization (MuMi) algorithm based on this idea has previously been applied for alanine mutational scanning to successfully determine positions critical for the functioning of Hsp70.^34^

Focusing on water interaction, which is crucial for assessing the mutational effects on protein and protein/ligand binding,^35^ literature indicates that stability predictors of mutational effects often use implicit water models or only solvent accessibility of the protein to model solvent effects.^4,5,7,9,10^ Although the correlation of predicted stability changes due to mutations is at a reasonable level compared to DMS stability experiments of GB1,^12,25,29^ the mutational perturbations on binding affinity are not rigorously investigated, given the difficulty of differentiating folding and binding from experimental results.^18,19^ While various computational tools predict binding affinity changes using implicit water models,^8,36−38^ the MuMi pipeline employs explicit water to investigate the molecular basis of binding computationally.^34^ Given that explicit water models achieve slightly better correlation,^39^ our method, utilizing the latest force field,^40^ should effectively capture the effects of binding changes caused by mutations.

In this study, we employ GB1 (Figure 1) and construct all possible single mutants of GB1 by using the MuMi scheme,^34^ as well as AlphaFold2 predictions of the mutants followed by minimization (AFMi).^41,42^ We also perform an extension of 1 ns-long MD simulation (MuMi-Dyn) for 14 positions (266 mutations) that are in close proximity to the binding interface. To form a solid basis for comparison, we conduct two 1 μs-long MD simulations of unbound and bound WT forms to establish the conformational dynamics sampled by the system at equilibrium. To understand the effects of single mutations on binding, we compare MuMi, MuMi-Dyn, and WT-MD simulations for unbound/bound forms. We find that the MuMi scheme generated structures carry enough information to explain the stability shifts due to point mutations. We also find that the hydrogen bond occupancies at the GB1–IgG-Fc interface is the main fingerprint of fitness. Since MuMi is an efficient method that has the right level of detail to predict the conformational shifts in proteins due to point mutations, our findings pave the way for developing machine learning methods that can locate the conformations on the fitness landscape.

In this manuscript, our key goal is to show if an efficient methodology that captures the essential changes introduced into the protein structure due to point mutations may be developed. We show that introducing point mutations into the protein structure followed by minimization in the correct solvent environment (i.e., MuMi) is a reliable and computationally efficient tool for predicting protein fitness landscapes, offering significant advantages over traditional methods. To achieve this goal, we first show the main ingredients that contribute to the binding stability. We then demonstrate that the proposed methodology predicts the experimental measurements across all mutations. Finally, to explain why such a simplified approach works, we make a full assessment of the physicochemical parameters that emerge in the generated structures, such as solvent accessibility, conservation scores, the role of residues involved in allosteric communication, and hydrogen bond distributions. Our findings serve to clarify molecular information carried by point mutations due to different situations which are of significance for molecular evolution, protein engineering and personalized medicine applications.

## Methods

### Simulations

A summary of all simulations conducted in this work is presented in Table 1 and are briefly described here. We use 1PGA and 1FCC coded crystal structures from Protein Data Bank^43^ (PDB) as unbound and bound forms of GB1 protein, respectively; the complex is displayed in Figure 1a and its sequence with the charged residues and mutated sequence positions are displayed in Figure 1b, top row. In the MuMi scheme, to construct 1064 single mutations (56 positions × 19 amino acid substitutions), we employ ProDy^15^ and Visual Molecular Dynamics (VMD).^44,45^ We then solvate the proteins in a cubic water-box that has a minimum distance of 10 Å to the protein in every direction so that a protein atom and another on its image are separated by at least 20 Å between. We use the explicit water model TIP3P^46^ for solvation, as explicit water models have been shown to achieve better results in investigating protein–protein binding.^39^ We add KCl salt to maintain the isotonic (0.15 M) concentration. Minimization of 10,000 steps is performed by using Nanoscale Molecular Dynamics (NAMD2) software, with CHARMM36 force-field.^40,47^ Alternatively, the prediction of protein structures belonging to single mutations is done by using ColabFold that uses AF as a base with a different MSA algorithm.^41,42,48^ We construct one model for each single mutation and use the same minimization procedure as in MuMi for the relaxation of structures in water; hence, this pipeline is named as AlphaFold2 and Minimization (AFMi). We also perform two duplicate 1 μs-long MD simulations for unbound and bound forms of WT GB1 at 310 K constant temperature and 1 atm constant pressure on the minimized WT structures. We again use NAMD2 and CHARMM36 force-field for the MD simulations.^40,47^ Furthermore, 14 amino acid positions (14 × 19 = 266 mutant structures) are selected from the residues that have high probability to be located at the binding region according to the MuMi results (see Results and Discussion). One ns-long MD simulation extensions for these mutant forms are conducted by using the same pipeline as MD simulations for the WT. However, due to excess energy occurrence after the insertion of mutant side chains, we are unable to perform MD simulations for D40L, E42H, E42M, E42F, and E42Y single mutant structures. By omitting these mutations, we produce 261 systems by using MuMi-Dyn method for further analyses.

**Table 1:** List of Simulations Conducted in This Work

abbreviation	methodology	number of mutations	length of each MD	total length of simulation
WT unbound MD	classical MD		1 μs (two replicates)	2 μs
WT bound MD	classical MD		1 μs (two replicates)	2 μs
MuMi unbound	mutation + minimization	1064		
MuMi bound	mutation + minimization	1064		
AFMi unbound	AF prediction + minimization	1064		
MuMi-Dyn unbound	MuMi + 1 ns MD relaxation	261	1 ns	261 ns
MuMi-Dyn bound	MuMi + 1 ns MD relaxation	261	1 ns	261 ns

### Analyses of Structures

Salt bridges, solvent accessible surface area (SASA) and hydrogen bonds are computed by VMD plugins.^44^ The hydrogen bond definition used is 3.0 Å donor–acceptor distance and 20° for the hydrogen bond angle. FreeSASA^49^ software is used for relative solvent accessibility (RSA) analysis to investigate the changes in solvent interaction profiles for each residue belonging to a protein structure. To calculate probability of being located at the binding region, we compute distances of all heavy (non-hydrogen) atoms between GB1 and its partner; then, if any two atoms of these two compounds are closer than 5 Å,^19^ the residue index on GB1 is saved as one occurrence of being located at binding region. All occurrences are calculated for WT structure, MuMi, MuMi-Dyn, and WT-MD simulations and displayed as probability density functions. Residues described as allosteric for GB1 (residues 9, 12, 14, 30, 33, 37, 39, and 56) have been taken from a recent study.^19^

## Results

### GB1–IgG-Fc Interface Is Maintained by Dynamical Hydrogen Bonds

The interface was defined by a RSA analysis which was previously used to categorize the surface and core residues of GB1.^19,30^ We calculate the RSA of each amino acid from the minimized WT structures and find that the RSA values and the ensuing definition of binding residues differ from the previous work where only the crystal structure was used for a similar analysis (Figure 1).^19^ This is because the crystal structures might represent artificial lattice restraints which are released upon minimization in the solvent environment. Residues near the binding region have propensity to interact less with water (Figure 1b), in particular, the solvent accessibility of residues 24, 27, 28, 31, 32, 35, 40, and 43 decreases in the bound form.

We next study the dynamics of unbound and IgG-Fc bound GB1 through duplicate MD simulations, each of 1 μs length. The RMSD and RMSF plots for these runs are presented in Figure S1. Both X-ray structures represent equilibrium states with less than 2 Å RMSD from the initial state of the system throughout the 2 × 1 μs length of the simulations. Increased RMSF values in the bound state for the residues distal to the binding interface reflects a general property of proteins.^50−52^ While this increased flexibility is usually accompanied by the rigidification of the binding site residues, in GB1 we find the latter effect to be minimal, because the interface is made up of secondary structural elements (Figure 1b).

**Figure 2:**
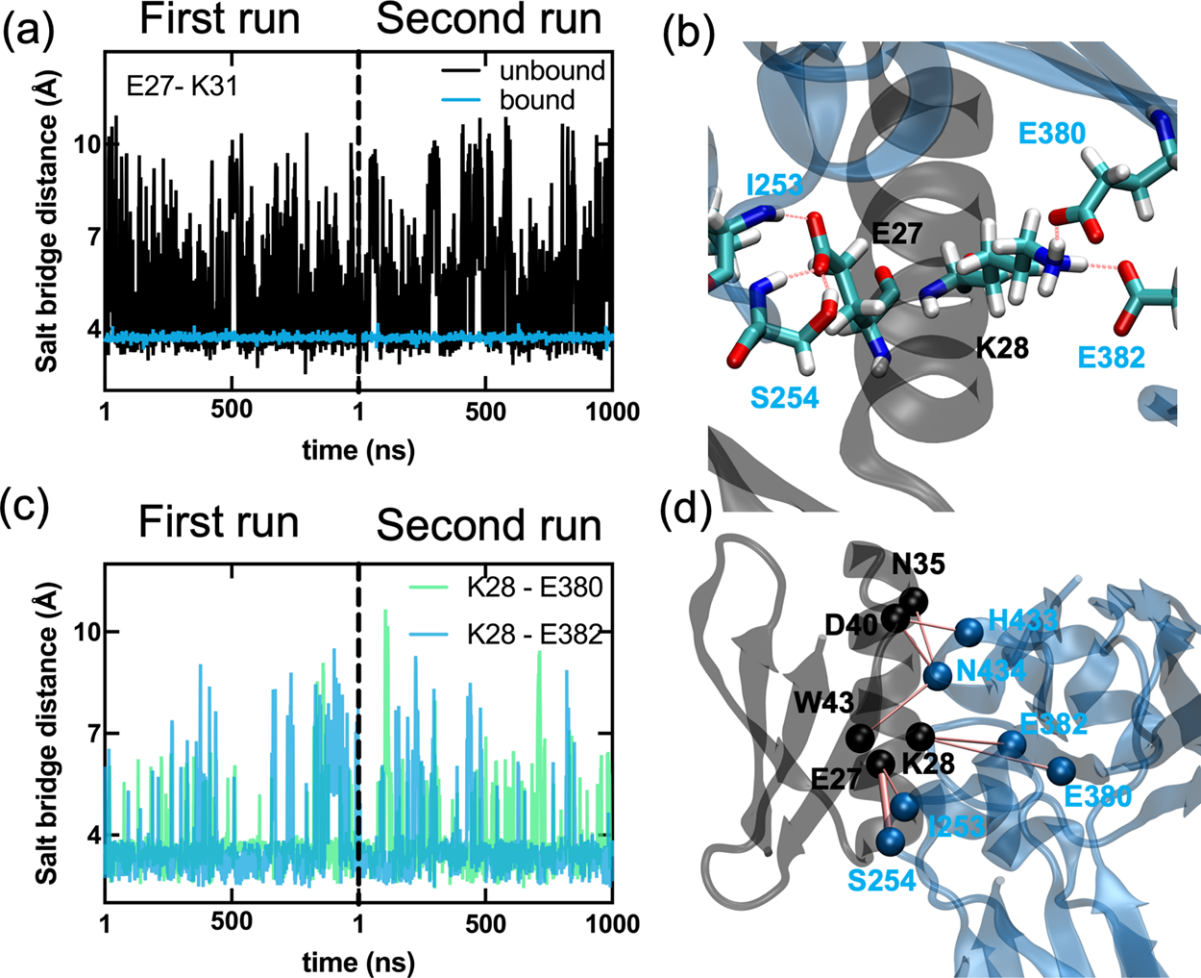
Key distances from WT-MD simulations; two independent 1 μs runs for each of the unbound and bound forms of GB1. The salt bridge distances are calculated based on center mass of both oxygen and nitrogen atoms. (a) E27–K31 distance in the apo structure is highly variable (black) but is completely stabilized upon binding (blue). (b) The coordination of salt bridges occurs between residues E27 and K28 of GB1 and residues I253, S254, E380, and E382 of IgG-Fc. Here, K31 stabilizes E27 with a salt bridge, leading to successful binding. The annotated residues are shown in licorice, and the salt bridges are depicted as dashed pink lines. (c) Stabilization at the interface is due to dynamical interactions of K28 from GB1 with E380 and E382 from IgG-Fc. (d) The network of interactions at the GB1 and IgG-Fc (see also Table 2).

A closer look at the interactions at the interface of GB1 upon binding lends further clues on the dynamics. According to the mutational scanning experiments of Olson et al.,^23^ the least mutable positions are E27, K31, and W43. All three of these residues are classified as “interface” residues due to the definition that any of their heavy atoms is within 5 Å of the binding partner.^19^ When effects of binding stability and folding stability are differentiated in these data, it was found that the stability of these residues all stem from the former.^18^ Of these, D27 and K31 are positioned at the binding interface at a distance which would enable them to form a salt bridge. However, our MD simulations reveal that the intramolecular D27–K31 is a loose salt bridge in the unbound form while it is completely stabilized upon binding (Figure 2a). This stabilization is caused by the strong K28–E380 and K28–E382 interactions at the interface (Figure 2b,c). Meanwhile, W43 performs a dual function. On the one side, with its bulky side chain, it partially occupies the hydrophobic core of the protein, being in contact with L5, A34, F30, F52, and V54. On the other side, it interfaces with the hydrophobic carbon tail of the K31 side chain close to the backbone, placing its NH_3_^+^ group in the correct orientation for the E27-K31 interaction mentioned previously. Thus, these residues are excellently positioned to stabilize the interface via the K28 interactions.

In Table 2, we list the 9 hydrogen bonds between GB1 and IgG-Fc with occupancies greater than 20% in the total of 2 μs WT-MD runs. We plot the dominant hydrogen bond network that achieves specific binding in Figure 2d.

**Table 2:** Hydrogen Bond Occupancies from 2 μs MD Simulations at the GB1-IgG-Fc Interface

residue identity for GB1	residue identity forIgG-Fc	hydrogen bond occupancy
E27-side chain	I253-backbone	39%
E27-side chain	S254-side chain	74%
E27-side chain	S254-side chain	43%
K28-side chain	E380-side chain	59%
K28-side chain	E382-side chain	54%
N35-side chain	N434-side chain	23%
D40-side chain	H433-side chain	38%
D40-side chain	N434-backbone	37%
W43-side chain	N434-side chain	44%

### MuMi-Generated Structures Encapsulate the Key Aspects of Binding Fitness Information

**Figure 3:**
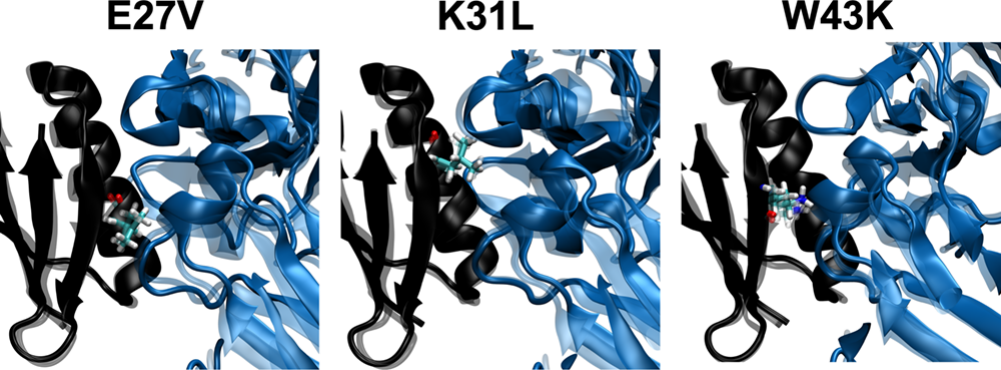
Effect of minimization for E27V, K31L, and W43K mutants. GB1 and IgG-Fc are shown in black and blue. Mutated residues are shown as licorice with atom coloring. Initial (mutated WT crystal structure, 1fcc PDB-coded) structure is illustrated as transparent; on the other hand, final minimized structures are illustrated in opaque colors. Heavy atom RMSD between the initial and final structures is 1.15, 1.07, and 1.11 Å for the respective MuMi replacements.

**Figure 4:**
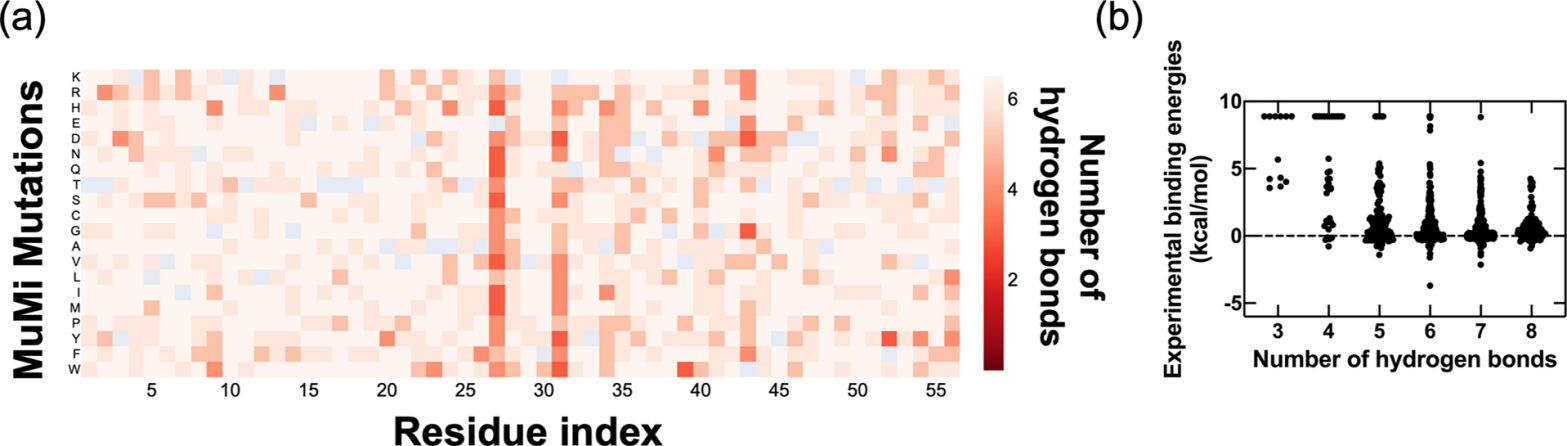
(a) Heatmap of number of WT intermolecular dominant hydrogen bonds for all MuMi-produced conformations. (b) The number of WT intermolecular dominant hydrogen bonds compared with the experimental binding energies is shown on the right. The experimental binding energies are capped at 9 kcal/mol and any mutation exceeding this amount of destabilization is reported through this value.^18^

We next seek if mere mutation of positions followed by minimization produces plausible structures that represent conformational shifts accompanying the side chain replacements. In Figure 3, we display three example mutations demonstrating that while the change introduced is local, long-range shifts in positions are achieved due to rearrangements in the soft degrees of freedom, which may lead to RMSD values exceeding 1 Å.^32,33,50^ In fact, we find that the plain count of the number of hydrogen bonds maintained at the interface following the MuMi scheme, where we display the scanning of the 56 positions with 19 amino acid substitutions in 1064 positions in Figure 4a, is a very good predictor of fitness for this system (Figure 4b).

We note the correspondence between the low stability caused by mutations at positions 27, 31, and 43 discussed in the previous subsection to be well described by the loss of the interface interactions. Interestingly, the K28 residue itself which establishes two stable hydrogen bonds in the WT (Figure 2b,c) is relatively more tolerant to single residue replacements which exactly follows the experimental findings,^19^ pointing to an intricate balance of interactions at the interface. We find that mutations on these specific residues have more impact than changes in amino acid type, such as charged and hydrophobic.

In addition to MuMi, we also employed FoldX^4^ to compute binding energies for bound GB1, as this method was used to compare experimental and computational results of GB1 stability. However, we were unable to achieve a significant correlation (*R*^2^ = 0) between the predicted and experimental binding energies^18^ (Figure S2). In Figure S2, a considerable decrease in stability change is detected for residues 27, 28, 31, and 43. These overlap with residues 27, 31, and 43, which are also emphasized by the DMS experiments.^18,23^ However, FoldX does not accurately predict the mutations that lead to significant changes, resulting in low correlations. This result may stem from the implicit water model used by FoldX, which was unable to detect surface mutations, considering that 33 residues (∼60% of all bound-GB1 residues) are surface residues (RSA > 25).^53^ Therefore, we believe that while predictions utilizing implicit solvation in their scoring functions may have general applicability, they lack the precision required for accurately predicting DMS results.

### MD Simulations and Mutational Scanning Provide Consistent Insights into the Functional Dynamics of the Protein at the Molecular Level

The relationship between conformational variability observed in μs-long MD simulations and that derived from mutational scanning studies is an aspect of interest in protein dynamics and function analysis. Thus, we analyze the overlap between the range of conformations sampled by the WT structure and those covered via single residue replacements. We analyze the 2 μs-long MD simulations both for the unbound and the bound forms of GB1 and compare our findings with MuMi by focusing on three factors: (i) the number of hydrogen bonds between GB1 and its partner; (ii) RSA differences of unbound/bound forms of GB1; and (iii) the probability of finding a residue at the binding region. The hydrogen bonds indicate robust interactions between GB1 and IgG-Fc. By interpreting solvent accessibility, we understand the water-mediated changes. Recent work has shown promise by classifying residues based on solvent accessibility without measuring binding energies.^19−22,35^

**Figure 5:**
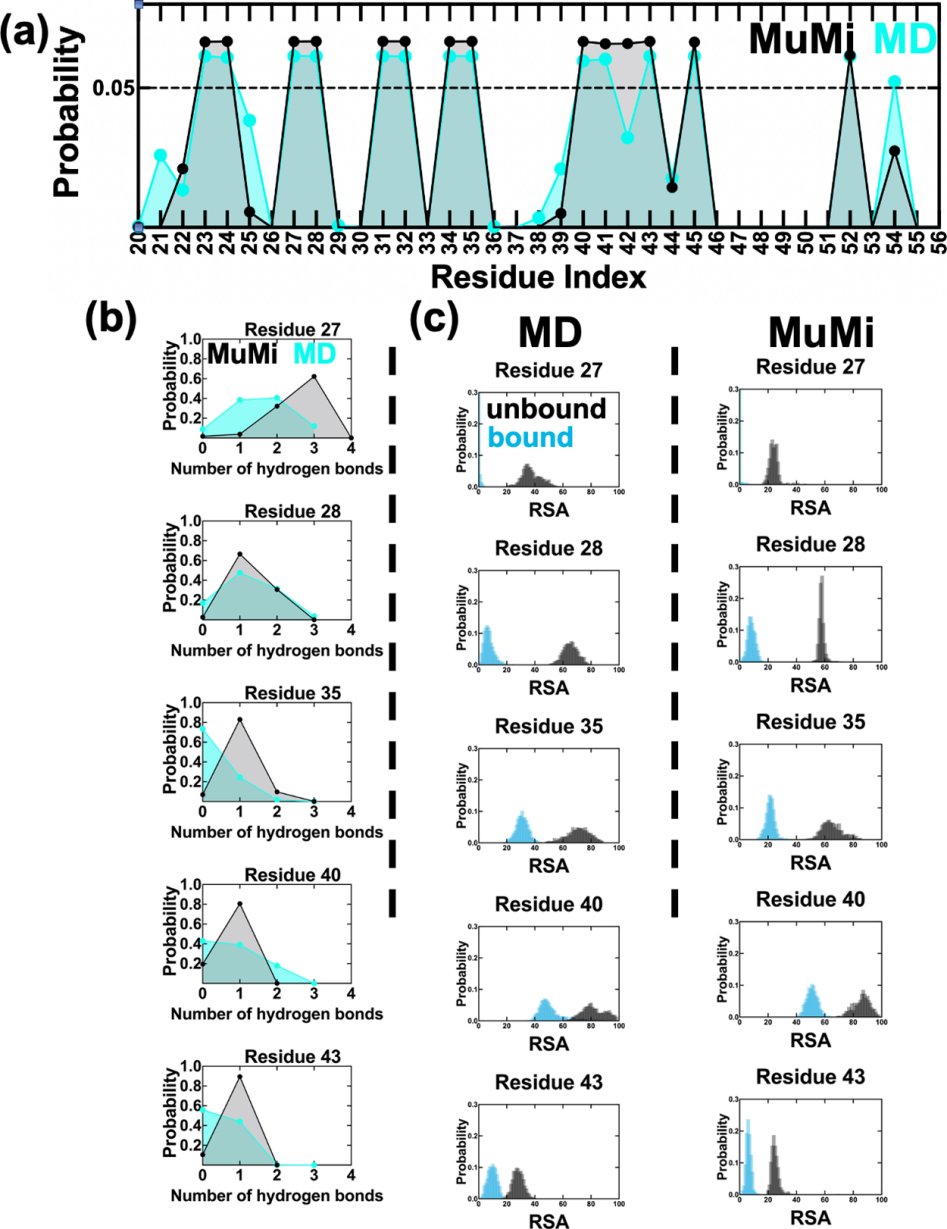
Assessment of GB1 binding by using MuMi and MD simulations. (a) Probability of residues being located at the binding region for mutations (MuMi) and MD simulations. Residues 1–19 are not shown due to insignificant probabilities. In MD results, residues 21, 25, 39, and 54 have higher propensity to stay at the binding region compared to MuMi, while residue 42 has lower probability. (b) PDFs of hydrogens bonds established between GB1 and IgG-Fc for residues listed in Table 2, calculated for MuMi and MD simulations. Residues tend to have a lower number of hydrogen bonds in MD simulations in contrast to MuMi. (c) PDFs of RSA are calculated for unbound/bound forms for the same residue set (see Figures S6 and S7 for PDFs of the complete set of residues of unbound/bound for MuMi and WT-MD).

Additionally, the assessment of GB1 residues located in the binding region is important to investigate the interactions between the protein and its binding partner.^19^ We find that for all single mutations, 14 positions have a higher probability of being located at the binding region than the WT (Figure 5a). We apply 1 ns-long MD simulations following MuMi for this subset, culminating 261 MuMi-Dyn simulations and confirm that the hydrogen bond distribution of 1 ns-long extension does not significantly differ from that of the WT-MD simulations.

In Figure 5b, we compare the probability distribution of the hydrogen bonds whose occupancies are listed in Table 2 with those obtained from MuMi (a comprehensive picture for all residues that display at least one hydrogen bond is in Figure S3). As a result of thermal fluctuations in the MD simulations, the total number of hydrogen bonds found at the interface averaged over the duration of the WT-MD is less than those found in the ensemble of minimized structures of MuMi (Figure S4a,b). Adding relaxations via 1 ns-long short dynamics on the mutants of the 14 residues located near the binding site releases some of the hydrogen bonds in the minimized structures and covers a range similar to that of WT-MD (Figure S4a,c).

As a result of the less tight binding at the interface, the protein SASA of unbound/bound conformations for WT-MD are higher than MuMi (Figure S5a,b). While this reflects into the RSA of individual residues (see examples in Figure 5b) MuMi predicts the local effects remarkably well and the coverage of single residue replacements is similar to that of the WT ensemble collected under dynamical conditions whereby large-scale motions and the impact of water are pronounced by the MD simulations. Probability density functions (PDFs) of all hydrogen bonds and RSA analyses for MuMi and WT-MD simulations are displayed in Figures S3, S6, and S7.

We find that SASA and total hydrogen bonds in MuMi-Dyn are similar to those from WT-MD, since the more dominant water interactions in MD simulations modulate these factors (Figures S4 and S5). Residues also generally have a lower number of hydrogen bonds as in WT-MD simulations in MuMi-Dyn method (Figures S4c and S8); however, residues 40 and 41 have lower probability of being located at the binding region compared to MuMi and WT-MD (Figures 5a vs S9). Considering that MuMi-Dyn was applied only on binding region residues, it is interesting to have a similar regime with the WT-MD simulations with these biased mutational perturbations. To compare water interaction of residues for MuMi, WT-MD, and MuMi-Dyn, we compare ⟨RSA⟩ and σ of RSA of each method for unbound/bound GB1.

### Similarity of Water Interaction Regime for MD Simulation of WT and the Single Mutations

Considering mutational insertions and their physical features, such as hydrophobicity, volume and charge, even with the drastic differences between the amino-acid type of WT and the mutation, the effects of single mutations may fall within stability margin.^30^ Therefore, we observe slight differences between the conformational space of WT and the single mutants through the MD trajectories and expect an overall similarity.

**Figure 6:**
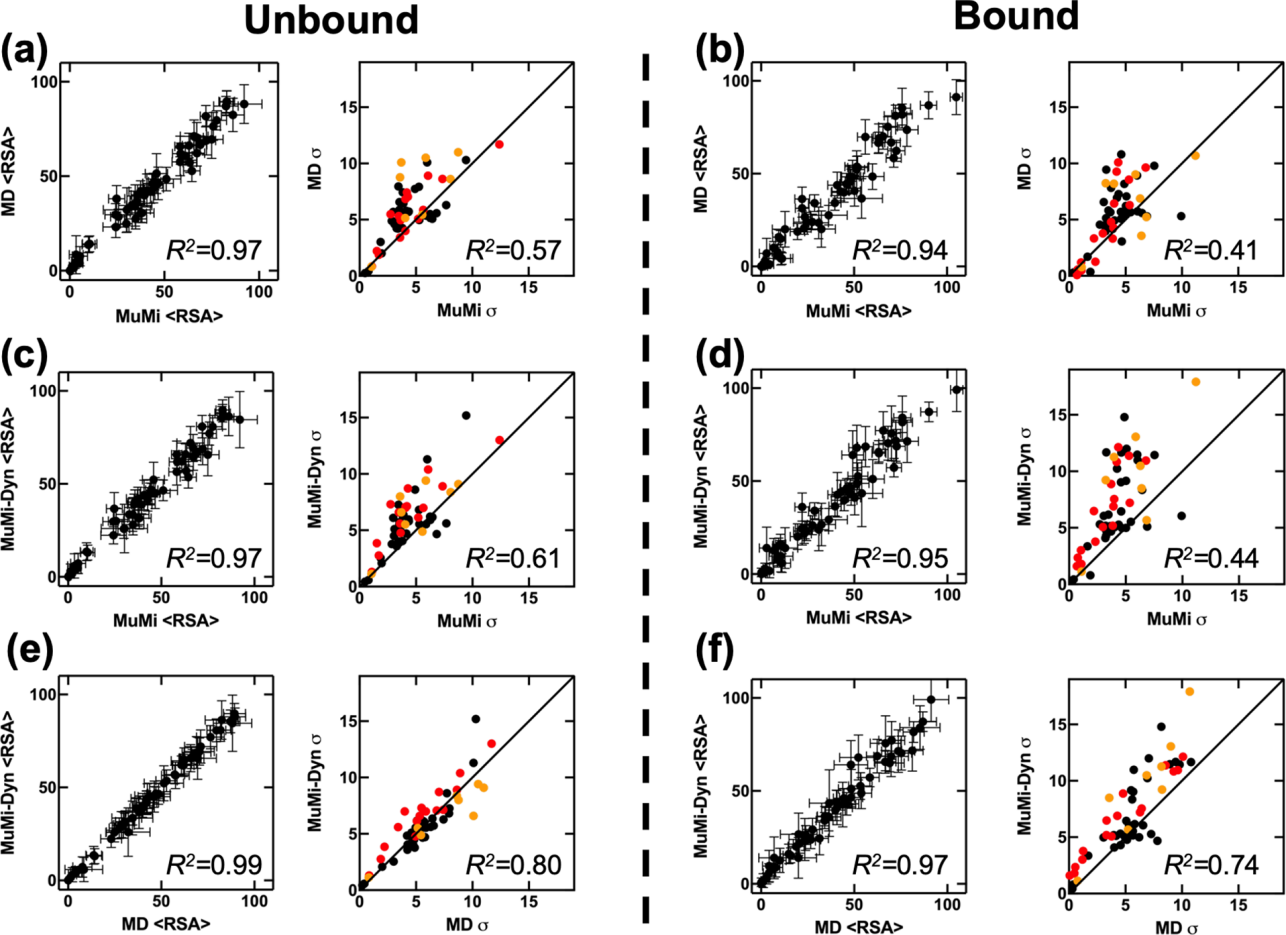
Comparison of ⟨RSA⟩ of unbound/bound form for MuMi, MuMi-Dyn, and WT-MD simulations. Allosteric residues and binding residues are indicated with orange and red, and *R*^2^ values are written on graphs. (a, b) The ⟨RSA⟩ and σ values of MuMi and MD simulations for unbound/bound. (c, d) The ⟨RSA⟩ and σ values of MuMi and MuMi-Dyn for unbound/bound. (e, f) The ⟨RSA⟩ and σ values of WT-MD and MuMi-Dyn simulations for unbound/bound (see Figures S6, S7, and S10 for PDFs of all residues by MuMi, MuMi-Dyn, and WT-MD simulations).

To this end, we compare RSA values of unbound/bound GB1 for MuMi, WT-MD and MuMi-Dyn, and find that the methods with MD simulations have wider RSA PDFs (Figures S6, S7, and S10). We find that not only the means, but also the standard deviations (σ) of PDFs are informative about the conformational effects of mutational perturbations. Overall similarity between ⟨RSA⟩ of MuMi, WT-MD and MuMi-Dyn indicates that water accessibility of residues is conserved through the single mutations (Figure 6). Note also that σ of RSA values are significantly similar for WT-MD and MuMi-Dyn results. (Figure 6e,f). The analyses of unbound and bound forms of GB1 does not differentiate the allosteric from the binding residues, indicating that GB1 achieves its function by modulating slight changes in the whole system, as also observed in PDZ3.^35,54^

Since point mutation structures can also be generated by alternative approaches, we next compare our MuMi and AF predicted structures (Figure S11). We performed MuMi and AFMi calculations for all single mutants and calculated the RSA for the final structures. The difference between MuMi and AFMi indicates that starting from the crystal WT structure is a sound method for constructing conformations representing single mutations to understand the mutational impact. In contrast, the conformational differences obtained via AFMi emerge from the varying MSAs (Figure S12) and the training of AF’s weights; therefore, they likely carry additional sources of variation.^55^

In the minimization process of MuMi, the effect of the local mutations is propagated to the instantaneous interactions. Conversely, the thermal fluctuations and solvent interactions are sampled via MD simulations leading to conformations reflecting the mutational characteristics more significantly. Interestingly, the biased mutations in the binding region and the coverage from WT-MD simulations are very similar in terms of ⟨RSA⟩ (Figure 6e,f). These results indicate that, although, there are exceptions like “deleterious” mutations, most of the single mutations modulate only the conformational space of protein structures. By fine-tuning the conformation space in varying fractions of time, the impact of single mutations on protein function/binding emerges. Thus, with larger sample sizes and longer MD simulations, it might be possible to investigate the conformational space of possible single mutations, without physically applying amino acid mutation on a protein structure.

## Discussion

DMS provides valuable insights into the structure–function relationships of proteins, the molecular basis of disease, and the mechanisms of protein evolution. It is widely used in protein engineering, drug discovery, and systems biology research to understand and manipulate protein function with high precision. Computational prediction of the effect of point mutations on the conformational landscape of proteins has the potential to overcome a crucial bottleneck in developing an understanding of the point mutational landscape of proteins.

To construct the single mutations of GB1, we compare MuMi and AFMi; the latter is an MSA-based structure prediction method,^42^ and the former is a crystal structure-based method.^34^ Overall, this comparison suggests that using the crystal WT structure as a starting point for modeling single mutations is a reliable method for understanding their impact. Conversely, the conformational variations observed with AFMi are due to differences in the MSAs and the tuning of AlphaFold’s weights, which may introduce additional sources of multiplicity. Hence, we find that, it is more prudent to perform mutagenesis by using a WT crystal structure.

The most striking finding of this work is that MuMi, a computationally feasible approach to generating computational DMS structures, provides enough information to reproduce the most important aspect contributing to the fitness of GB1, i.e., binding energy (Figure 4). While we find a single feature defined by the number of hydrogen bonds at the interface already correlates well with the fitness, this observation paves the way to using MuMi-generated structures as the input to machine learning algorithms to predict fitness landscapes through computational DMS.

The binding of GB1 is further scrutinized by focusing on three factors, that are hydrogen bonding of GB1 to IgG-Fc, probability of being located at the binding region and RSA analysis. Our results comply with a previous study,^24^ where residues 39, 40, and 41 were experimentally shown to have important effects on binding of GB1. The effects of single mutants are mostly subtle, and the conformation distributions do not discriminate between allosteric and binding residues. By employing these three analyses, we describe the most important elements of binding. Our findings serve to clarify molecular information carried by point mutations due to different situations which are of significance for molecular evolution, protein engineering, and personalized medicine applications.

## Data Availability

The code used for MuMi scheme can be found at https://github.com/midstlab/MuMi_scheme. All raw data and analyses codes are deposited at https://zenodo.org/records/11399775.

## Supporting Information Available

RMSD and RMSF results of MD simulations for unbound/bound GB1; FoldX energies for ΔΔ*G* of binding; probability of hydrogen bonds between GB1 and the partner for MuMi and WT-MD simulations; probability distribution of total hydrogen bonds between GB1 and IgG-Fc; probability distribution of SASA for unbound/bound GB1; probability of RSA of unbound/bound GB1 for MuMi; probability of RSA of unbound/bound GB1 for WT-MD simulations; probability of hydrogen bonds between GB1 and the partner for MuMi-Dyn; probability of being located at the binding interface for MuMi-Dyn; probability of RSA of unbound/bound GB1 for MuMi-Dyn; comparison of MuMi and AFMi results for unbound GB1; MSA depth for predicted AF structures; RSA probability distributions from MuMi and AFMi (PDF)
