# Supplementary material for: Deciphering GB1’s Single Mutational Landscape: Insights from MuMi Analysis
Source: J Phys Chem B. 2024 Aug 8;128(33):7987–96. doi: 10.1021/acs.jpcb.4c04916 (PMC11671028; doi:10.1021/acs.jpcb.4c04916)
Supplement: Supplementary file 1 — jp4c04916_si_001.pdf [file jp4c04916_si_001.pdf]

Supporting Information

Deciphering GB1's Single Mutational Landscape: Insights from MuMi Analysis

Tandac F. Guclu*, Ali Rana Atilgan, Canan Atilgan

Faculty of Natural Sciences and Engineering, Sabanci University, Tuzla, 34956, Istanbul, Turkey.

***Correspondence:** Tandac Furkan Guclu, Faculty of Natural Sciences and Engineering, Sabanci University, Tuzla 34956 Istanbul, Turkey, E-mail: tguclu@sabanciuniv.edu

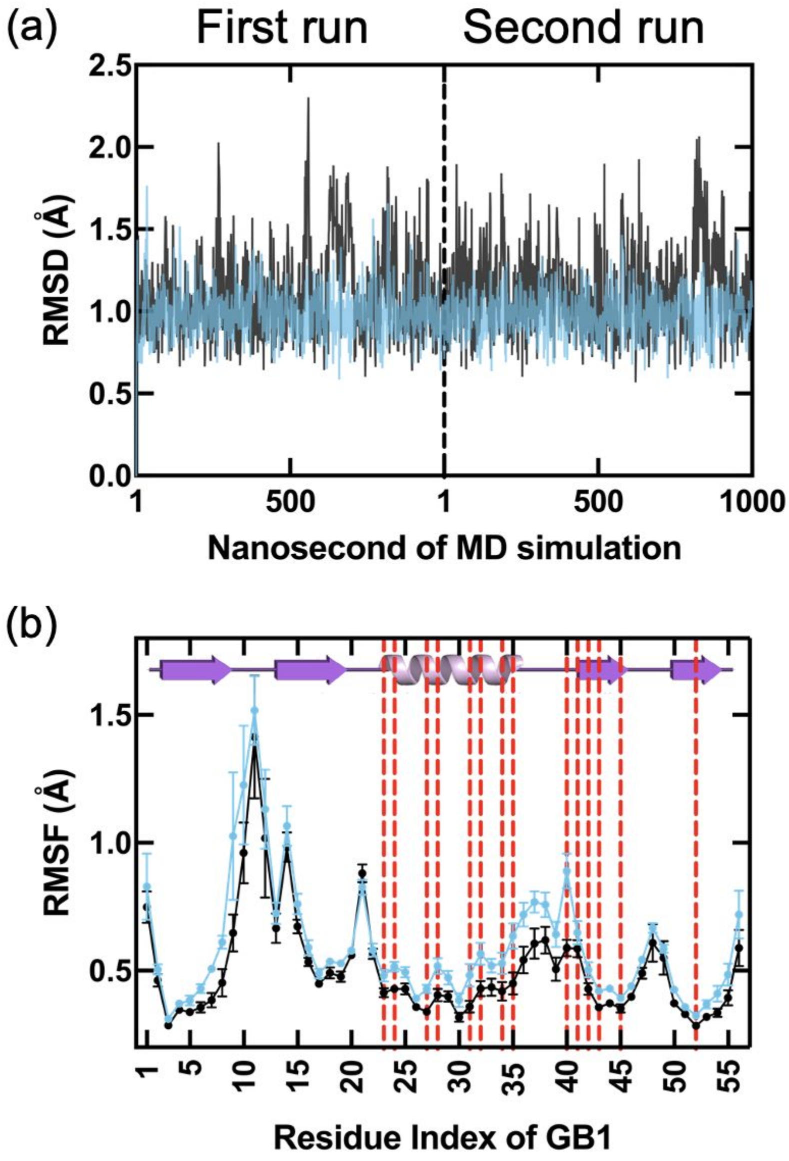

Figure S1 | (a) Root mean squared deviations (RMSD) from the MD simulations of the unbound (black) and bound (blue) forms of the WT GB1. The two runs are appended with the dashed vertical line separating them. Reference structure is the respective minimized WT crystal structure. **(b)** Root mean squared fluctuations (RMSF) results for the same runs. Averages are taken over eight, 250 ns-long batches; mean and standard deviations are shown on graph. The binding residues of the minimized WT complex are indicated with dashed red lines.

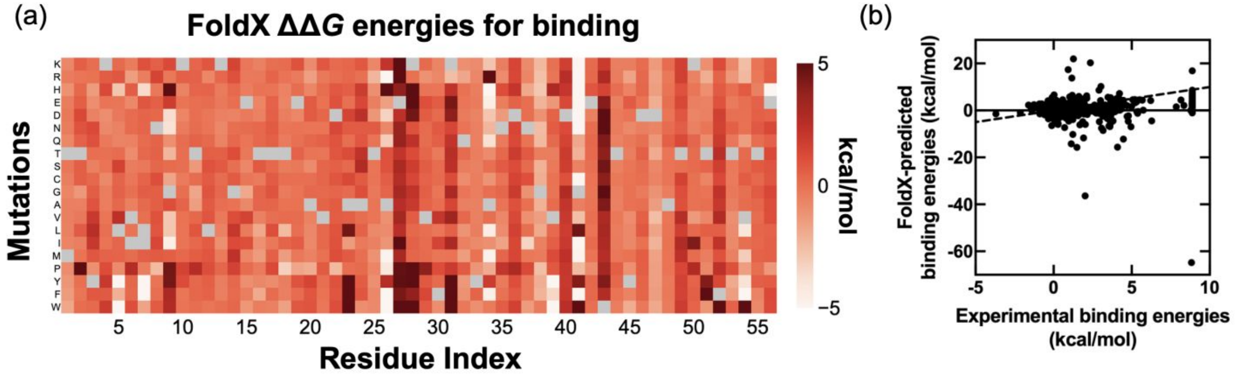

Figure S2 | (a) FoldX[1] energies for ΔΔ*G* of binding were calculated by taking the difference in mutation energies between the bound and unbound forms of GB1. The energies for the unbound/bound forms were calculated using the PositionScan tool of FoldX. With respect to the WT GB1, favorable mutations are shown in white, and unfavorable mutations are shown in red. Omitted mutations (self-mutations) are annotated in gray. Please note that the differences in sequences of the unbound and bound forms lead to two omitted mutations for four positions. To focus on slight variations, a heatmap is visualized for energy ranges between -5 and 5 kcal/mol. **(b)** The comparison of experimental and FoldX predicted energies is shown, with the line of identity visualized by a dashed line. These predicted energies do not correlate with the experimental binding energies (R^2 = 0).[2]

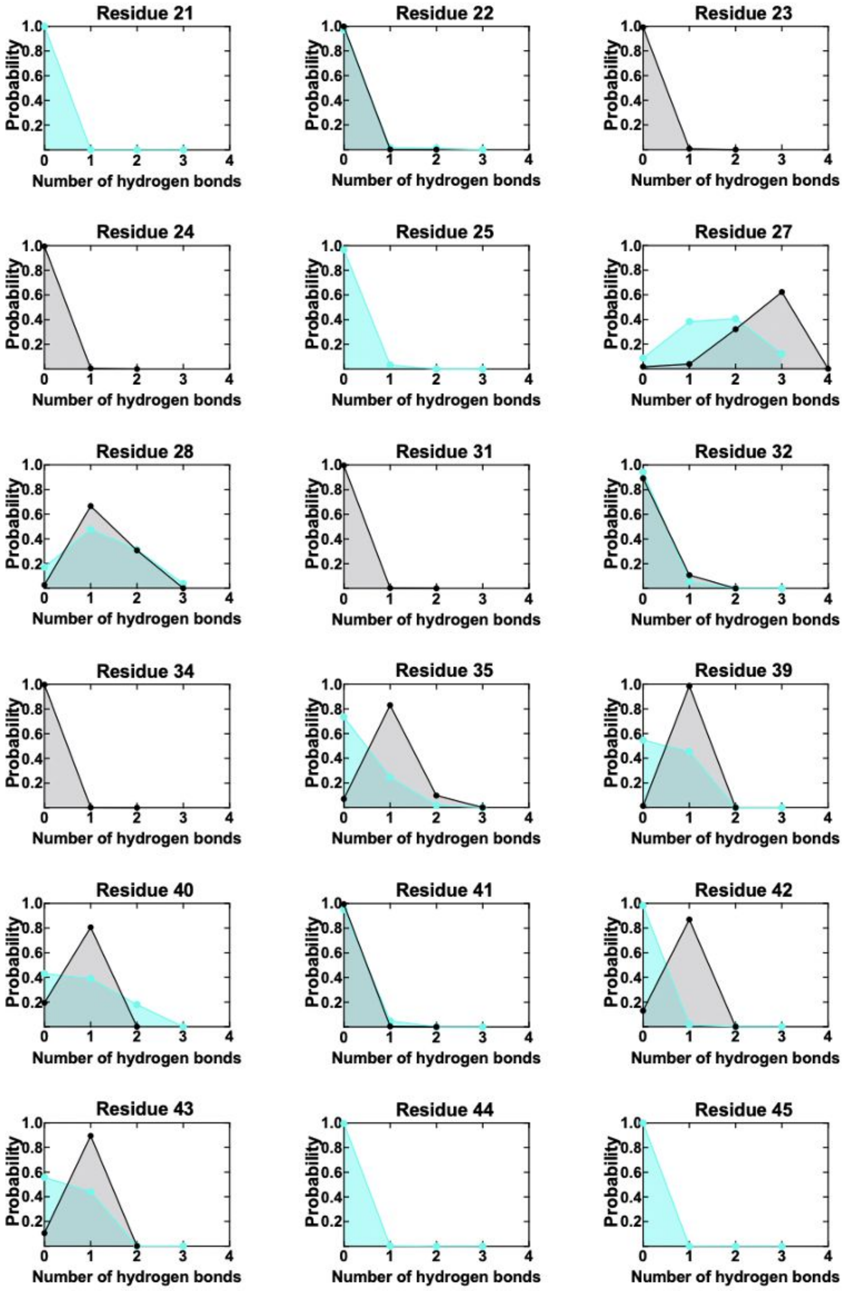

Figure S3 | Probability of hydrogen bonds between GB1 and the partner for MuMi (in black) and WT MD simulations (in cyan). All residues having even a single occurrence of hydrogen bonds are shown.

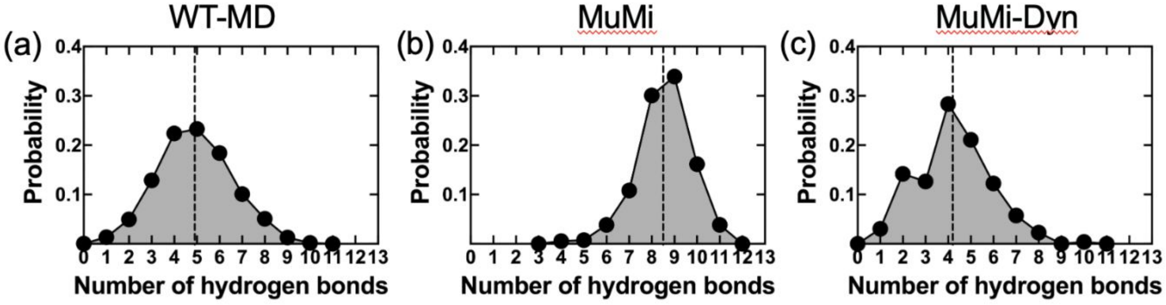

Figure S4 | Probability distribution of total hydrogen bonds between GB1 and IgG-Fc for, **(a)** 2 µs-long MD simulations; **(b)** MuMi; and **(c)** MuMi-Dyn. Mean values are indicated by the vertical dashed lines. In minimized structures **(a)**, the number of intermolecular hydrogen bonds varies between 3-12 with an average of 8.5 bonds.

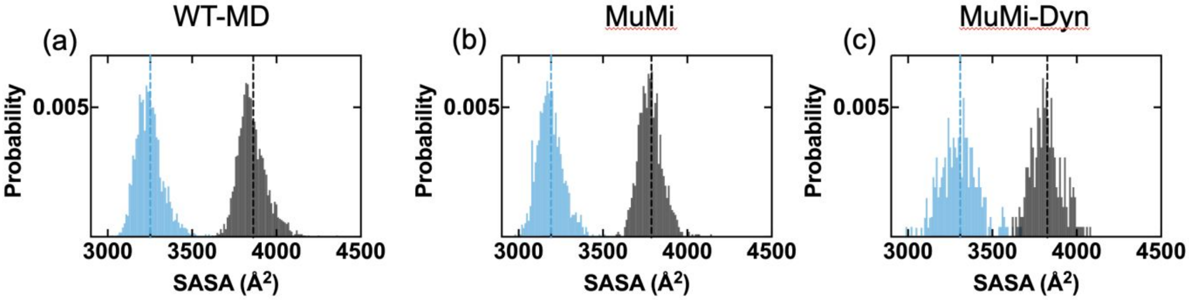

Figure S5 | Probability distribution of SASA for unbound (black) and bound (blue) GB1 for, **(a)** 2 µs-long MD simulations; **(b)** MuMi; and **(c)** MuMi-Dyn. Mean values are indicated by the vertical dashed lines. SASA values of minimized WT for unbound and bound forms are 3698 and 3239 Å^2, respectively. After the 1 ns MD extension of WT form, SASA values increase to 3800 and 3388 Å^2 in unbound and bound forms, respectively. In bound form, solvent accessibility is decreased for all methods.

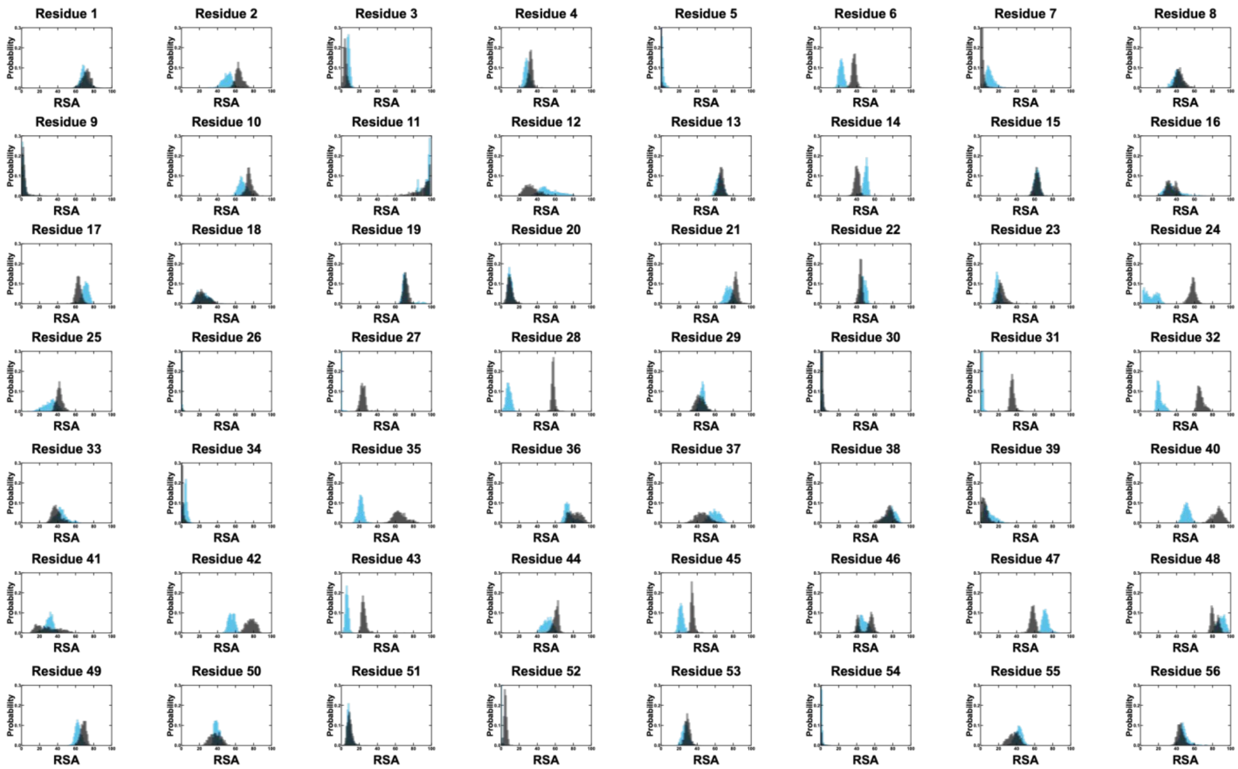

Figure S6 | Probability of RSA of unbound (in black) and bound (in blue) GB1 for MuMi. Probabilities are displayed for 56 residue positions.

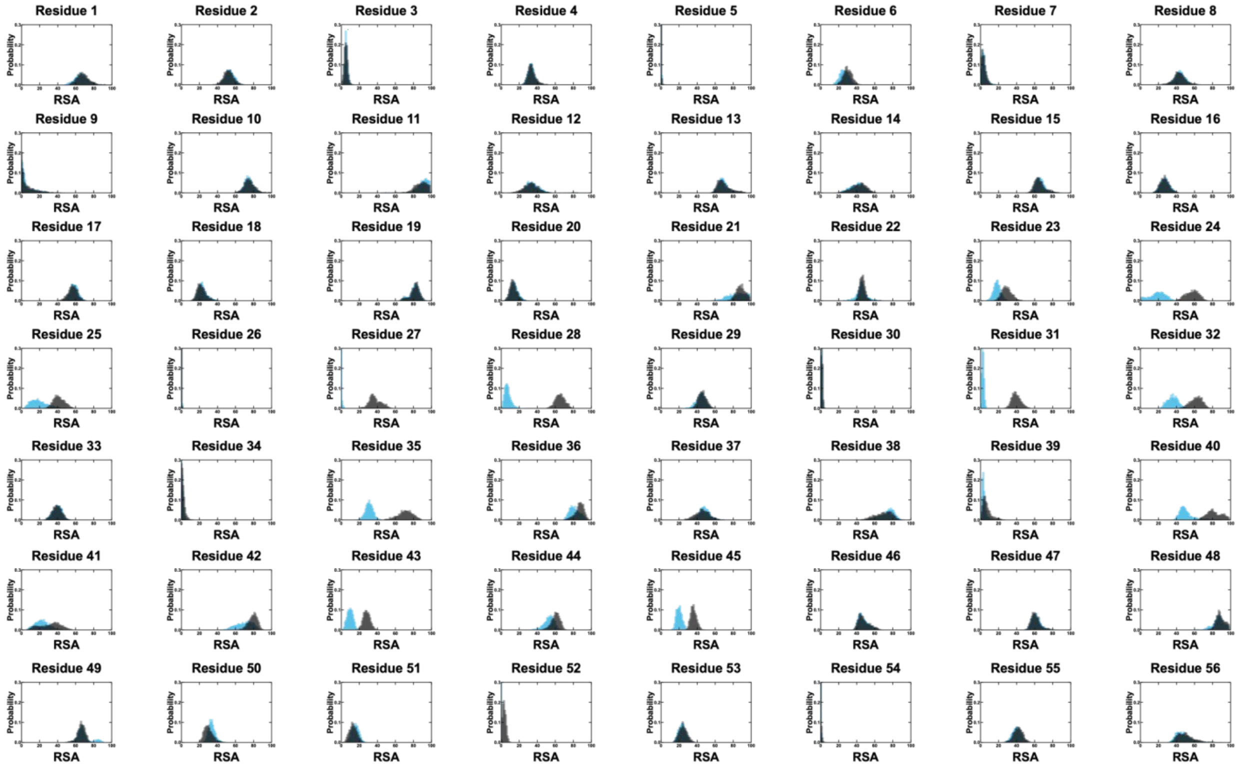

Figure S7 | Probability of RSA of unbound (in black) and bound (in blue) for WT-MD simulations. Probabilities are displayed for 56 residue positions.

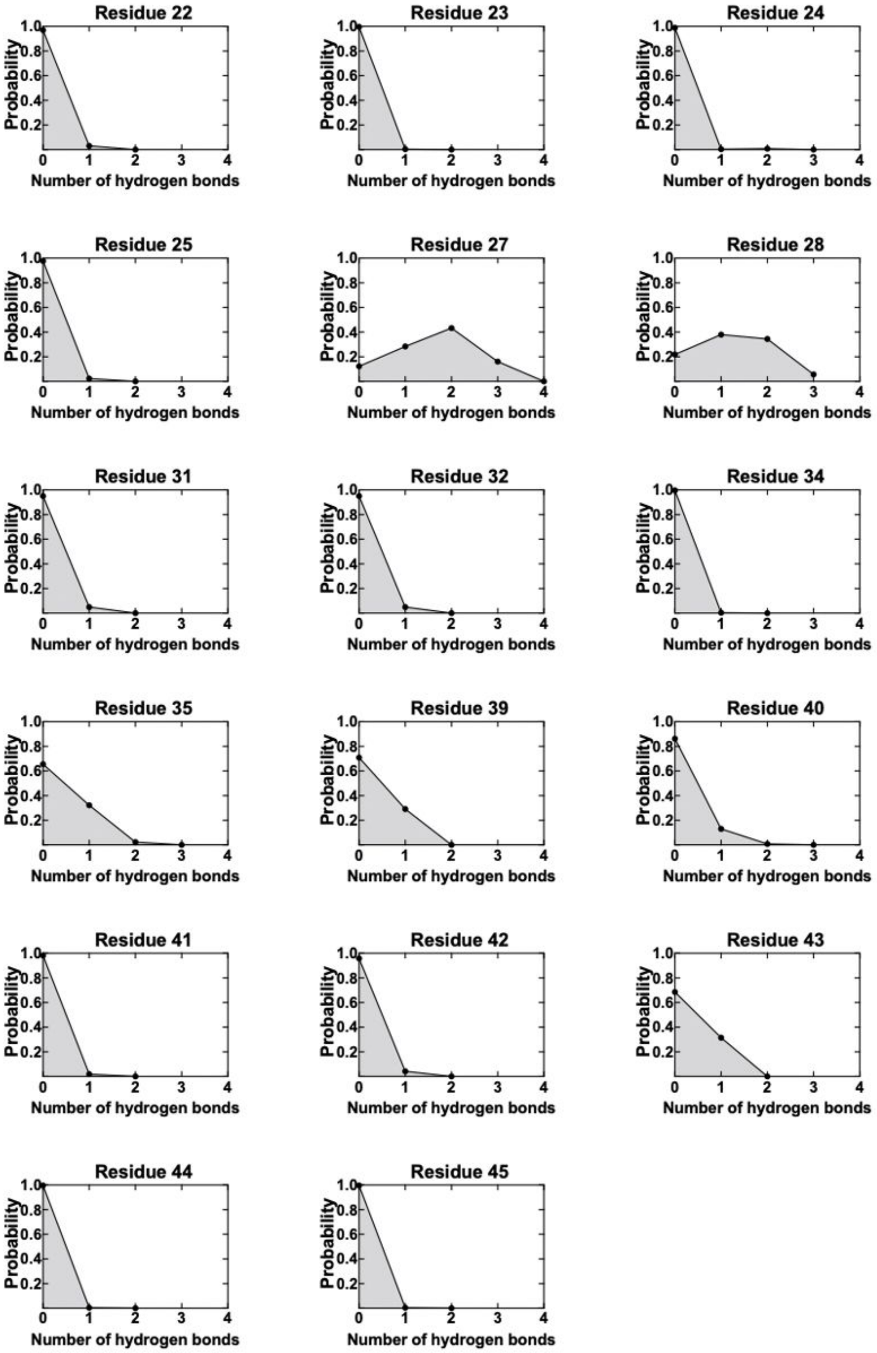

Figure S8 | Probability of hydrogen bonds between GB1 and the partner for MuMi-Dyn. All residues having even a single occurrence of hydrogen bonds are shown.

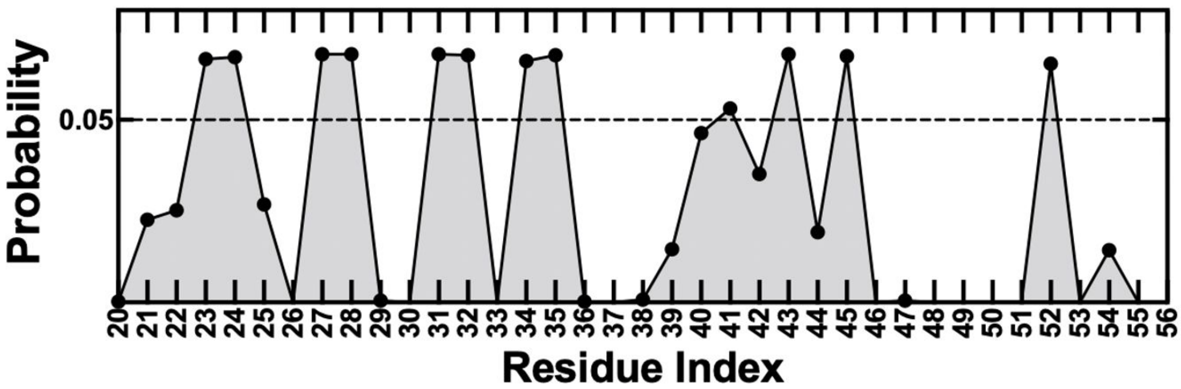

Figure S9 | Probability of being located at the binding interface, calculated for MuMi-Dyn. Compared to MuMi and WT MD simulations, residues 40 and 41 have lower probability.

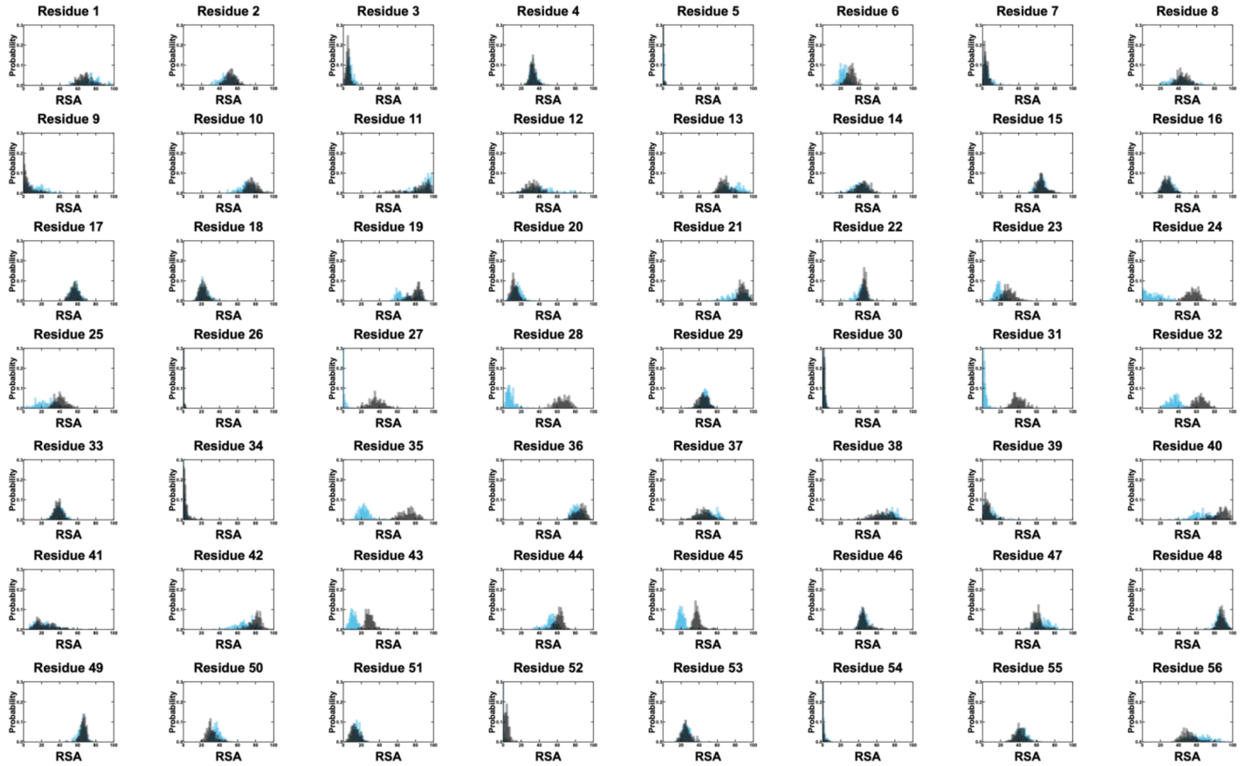

Figure S10 | Probability of RSA of unbound (in black) and bound (in blue) for MuMi-Dyn. Probabilities are displayed for the 56 residue positions.

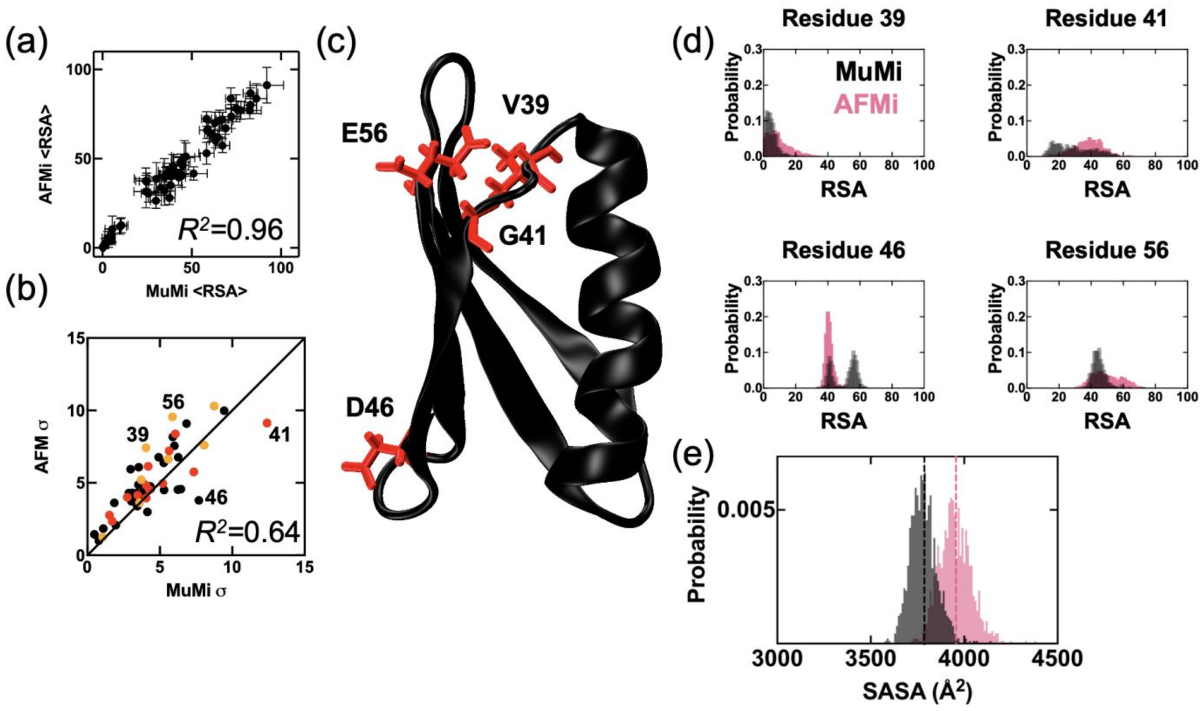

Figure S11 | (a) RSA results of all-possible single mutation conformations (56 positions × 19 amino acid substitutions) generated by MuMi and AFMi schemes for unbound GB1. ⟨RSA⟩ shows the average effect of mutational perturbations to that amino acid position through the single mutations. ⟨RSA⟩ results of single mutations for MuMi and AFMi are also very similar, which indicates that mutational insertions to the crystal structure and AF-predicted mutant conformations are very similar on average. **(b)** Comparison of standard deviation (σ) of RSA for MuMi and AFMi showing the importance of probability distributions over averaging to scrutinize the effects of single mutations. Binding region residues from the minimized WT complex are shown in red, and allosteric residues from a previous study[3] are shown in orange. The comparison of their variance shows that there are significant differences in the predictions for positions 39, 41, 46 and 56 (Figure S11b-c). Residue 41 is known for its position at the binding interface (Figure 1b) without taking part in the hydrogen bond network of Table 2. Residues 39 and 56 were reported as allosteric.[3] **(c)** Outliers from (b) are visualized on the WT unbound-GB1 structure. **(d)** Probability density functions (PDFs) of RSA values of single mutations of outlier positions. The RSA probability distributions for these residues are displayed in Figure S11d (see Figure S13 for all positions). Positions 39 and 56 sample a wider range solvent exposure

in AFMi predictions. Conversely, residue 46 has two states of different solvent exposure in MuMi structures, while it occupies only one state in those from AFMi. Position 41 samples a variety of states in both approaches, but more so in MuMi than AFMi; since this is a glycine in the WT and is located on a loop, perturbations cause significant impact on its conformations. **(e)** Total SASA of the proteins from AFMi (pink) and MuMi (gray). The total SASA distribution of AFMi generated structures is shifted into larger values than those from MuMi; i.e. AF predicts slightly expanded structures for GB1. In general, the difference between MuMi and AFMi indicates that, to construct conformations representing single mutations, starting from the crystal WT structure is a sound method to understand the mutational impact. In contrast, the conformational differences obtained via AFMi emerge from the varying MSAs (Figure S12) and training the weights of AF; they are therefore likely carry additional sources of variations.[4]

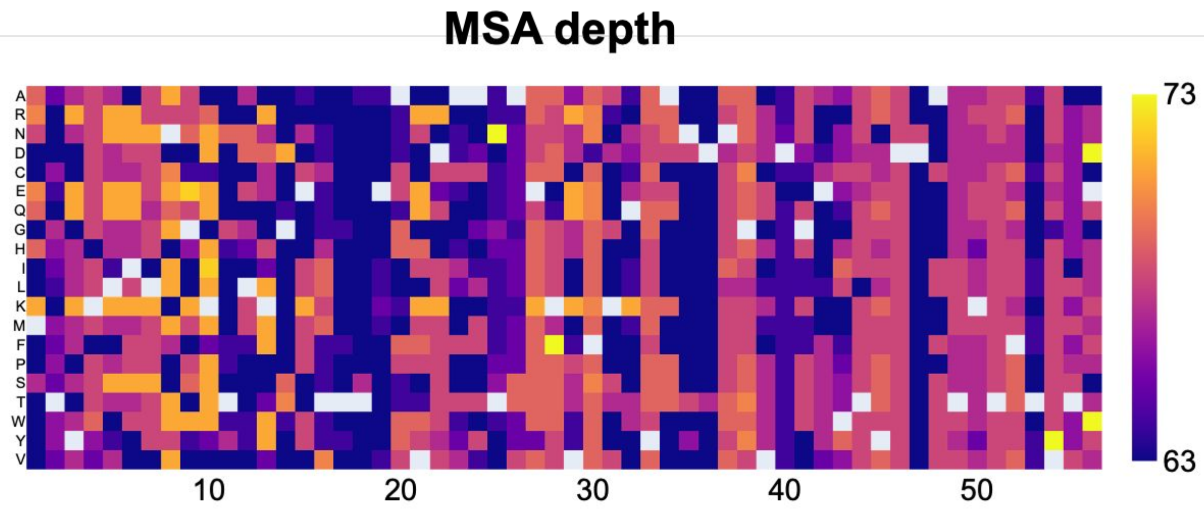

Figure S12 | Multiple sequence alignment (MSA) depth for predicted AF structures. Dark blue and yellow correspond to 63 and 73, respectively. White squares indicate the self-mutations, which are not computed. MSA depth shows the number of unique protein sequences in an MSA that is used to predict structures. Difference between MSAs causes the variation between the predicted structures. MSA coverage has also been calculated, and the values change between 0.97–0.98.

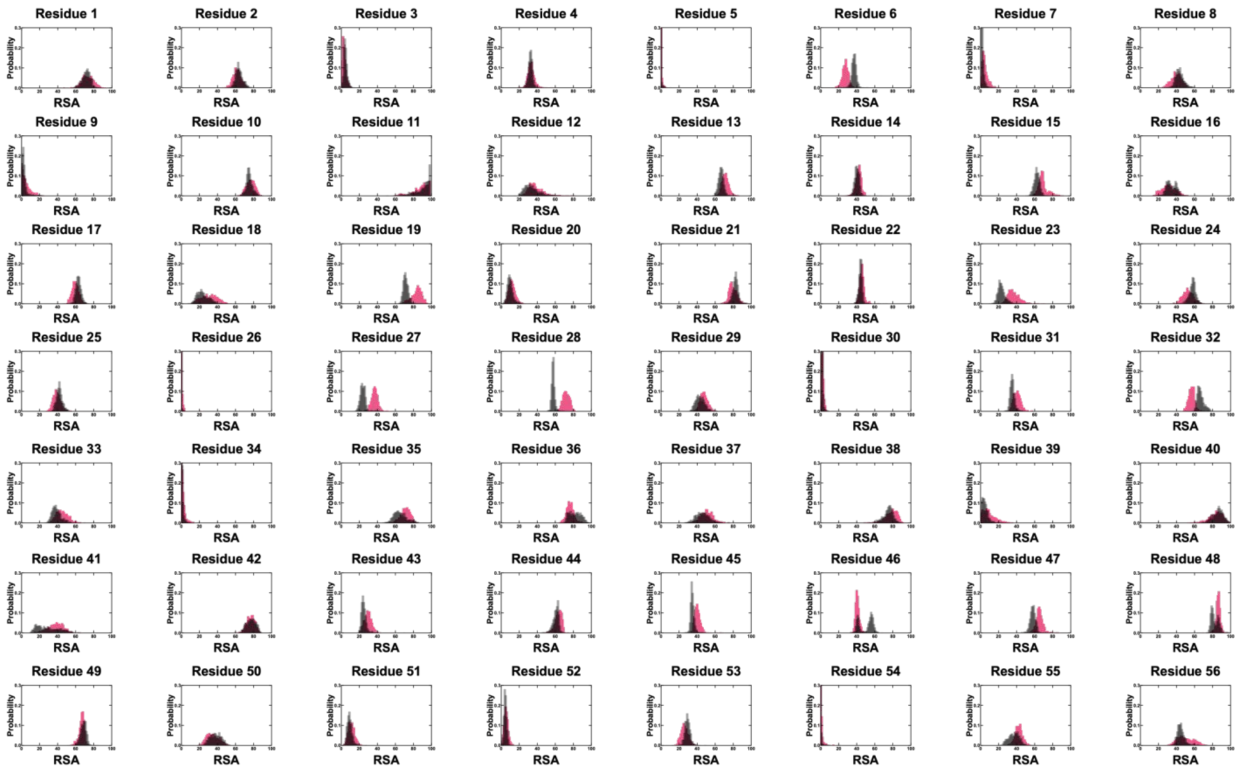

Figure S13 | RSA probability distributions from MuMi (in black) and AFMi (in pink) for all 56 positions for unbound GB1.

1. Schymkowitz, J. *et al.* The FoldX web server: An online force field. *Nucleic Acids Research* **33**, 382–388 (2005).
2. Otwinowski, J. Biophysical inference of epistasis and the effects of mutations on protein stability and function. *Molecular Biology and Evolution* **35**, 2345–2354 (2018).
3. Faure, A. J. *et al.* Mapping the energetic and allosteric landscapes of protein binding domains. *Nature* **604**, 175–183 (2022).
4. del Alamo, D., Sala, D., Mchaourab, H. S. & Meiler, J. Sampling alternative conformational states of transporters and receptors with AlphaFold2. *eLife* **11**, e75751 (2022).
